# Impact of Affirmation Relaxation and Back Massage on Depression, Anxiety, and Stress in Mothers Who Have Undergone Caesarean Section

**DOI:** 10.7759/cureus.81401

**Published:** 2025-03-29

**Authors:** Tity Jose, Hephzibah Kirubamani

**Affiliations:** 1 Obstetrics and Gynaecological Nursing, Saveetha College of Nursing, Saveetha Institute of Medical and Technical Sciences, Saveetha University, Chennai, IND; 2 Obstetrics and Gynaecology, Saveetha Medical College and Hospitals, Saveetha Institute of Medical and Technical Sciences, Saveetha University, Chennai, IND

**Keywords:** affirmation relaxation, anxiety, back massage, depression, stress

## Abstract

Introduction

The postpartum psychological well-being and quality of life of mothers after caesarean section are poor when compared to mothers following vaginal delivery. Therefore, the study aimed to evaluate the impact of a combination of back massage and affirmation relaxation technique on depression, anxiety, and stress experienced by mothers who had undergone caesarean section.

Methodology

This interventional study used a pretest-posttest control group design and was quantitative in nature. Twenty-four caesarean section mothers, with 12 in the experimental group and 12 in the control group, were chosen by a purposive sampling technique at Fatima Hospital, Mau, Uttar Pradesh, India, from July 1, 2022, to August 14, 2022. A structured questionnaire on sociodemographic data and a standardised tool, DASS-21 (Depression, Anxiety and Stress Scale, 21 items), were used to gather the data. Data collection was done from the experimental and control groups on the 3rd and 6th post-operative days. The experimental group was given a back massage and affirmation relaxation four times a day for 10 minutes each on the 4th, 5th, and 6th post-operative days. The control group mothers received routine health assessment and health education on postnatal care and breastfeeding. Descriptive and inferential statistics were used to analyse the data using IBM SPSS Statistics for Windows, Version 26 (Released 2019; IBM Corp., Armonk, New York). The analysis of categorical data was done using frequency and percentage distribution, whereas the analysis of continuous variables was done using mean and standard deviation. The Wilcoxon signed rank test and the Mann-Whitney U test were employed to evaluate the intervention's efficacy. The significance level was set at p < 0.05.

Results

A total of 24 mothers who have undergone caesarean section participated in the study. Baseline characteristics such as age, type of family, education, parity, type of LSCS, gender of the newborn, birthweight of the newborn, and timing of initiation of breastfeeding were homogeneously distributed in both experimental and control groups. Before intervention, the mean depression score of both the experimental group (8 ± 2.09) and the control group (8.50 ± 2.28), the mean anxiety score of the experimental group (11.67 ± 3.06) and the control group (11.50 ± 2.97), and the mean stress score of the experimental group (10.33 ± 3.17) and the control group (11.00 ± 2.63) were comparable. After intervention, there was a significant reduction in the maternal stress score in the experimental group (6 ± 2.83) compared to the control group (8.67 ± 2.31), which was statistically significant at p < 0.05. The anxiety score was also reduced after intervention in the experimental group (6.50 ± 3.53) compared to the control group (9.17 ± 1.99), which was nearly significant (p = 0.052). The mean depression score between the experimental group (6.50 ± 1.93) and the control group (7 ± 2.49) was not statistically significant (p > 0.05).

Conclusion

The study findings highlight the potential benefits of affirmation relaxation and back massage for improving the psychological well-being of postpartum women who have had caesarean sections. The advantages of this mother-friendly, cost-effective method should prompt hospitals and health centres to incorporate it into standard postpartum care. Nursing students should also be taught about it as part of their midwifery education.

## Introduction

Pregnancy and related events are very significant in the life of a woman, during which life goes through tremendous changes physiologically, biologically, physically, emotionally, and psychologically. The birth of a baby naturally brings joy to the mother and her family. But for a few, pregnancy and childbirth are stressful due to various factors and can alter their psychological and emotional well-being [1].

Approximately 10-30% of women experience postpartum depression worldwide [2]. A meta-analysis of 38 Indian studies reported that about 22% of Indian mothers suffer from postpartum depression. The findings further explain that in the southern region, the pooled prevalence was 26%; in the eastern region, 23%; in the south-western region, 23%; and in the western region, 21% of mothers experience postpartum depression. The lowest prevalence was in the northern region (15%) [3].

Postpartum depression may give rise to emotional, behavioural, and psychiatric problems, and hyperactivity in children born to such mothers. Women typically feel stressed, sad, lonely, or fatigued after giving birth [4]. Postpartum depression can affect any woman and is most common among young mothers, high-risk pregnancies, and primipara mothers [5].

Caesarean sections are one of the most common surgical procedures worldwide. However, they may cause mental and physical complications in the mother [6]. Physical complications associated with caesarean section include intra- and post-operative bleeding, infections, intra-abdominal adhesions, deep vein thrombosis and pulmonary embolism, hysterectomy due to uncontrollable bleeding, brain hazards, increased risk of wound dehiscence, maternal death, etc. [7].

Psychological issues affecting mothers who have had caesarean sections include anxiety and stress [8]. Stress is the body's physiological, mental, and emotional reaction to the pressures of daily living, and anxiety is one of its by-products. The experience and understanding of stress by an individual depend on his or her preconceived perceptions, existing coping mechanisms, and their interactions with the environment, rather than the cause of stress itself [9]. Coping strategies differ from person to person. Stress-bearing capacity also differs.

Anxiety is an extremely unpleasant emotion that manifests as intense fear, distress, and doubt about unknown factors [8]. The sympathetic, parasympathetic, and endocrine systems are stimulated by the stress and anxiety associated with surgery. This can lead to symptoms such as palpitations, elevated blood pressure, shortness of breath, tremors, sweaty palms, decreased salivary secretion, dry mouth, increased gastrointestinal motility, and urinary incontinence [10]. Stress affects both the pregnant woman and her fetus. Maternal stress causes increased adrenaline release, which lowers blood supply to the placenta and fetus [11]. Stress inhibits oxytocin production and may increase postpartum haemorrhage [12].

Emotional discomfort during the postpartum period in mothers who have given birth by caesarean section is exacerbated by a variety of factors. Hormonal changes, pre-existing psychological problems, pain at the site of incision, unwanted or unsafe pregnancy, a difficult birth process, family conflicts, lack of social assistance, inadequate support from the medical staff, obstetric complications such as diabetes, pre-eclampsia, low birth weight, low socio-economic status, anxiety and depression during pregnancy, stress caused by caring for and breastfeeding the baby, and stress related to hospitalisation are all recognised as predisposing factors [13, 14]. Postpartum stress can lead to poor maternal and child health outcomes and the development of psychological problems. Factors that influence stress levels during this period include any form of pre-existing stress, baseline mental health status, the mother's past mental health issues, lack of encouragement from the spouse, birth of a daughter, illness or death of a child, substance abuse by the husband, high parity, low educational level of the mother, and other associated risk factors.

Psychological distress can cause long-term impacts that may adversely affect the life of mothers, children, and families if it is not recognised and not treated, leading to cognitive, emotional, and behavioural problems in the child [14].

Stress-bearing capacity differs from person to person. Research has shown that in the midst of the COVID-19 outbreak, the prevalence of postpartum stress increased, along with symptoms of postpartum depression, anxiety, and perceived stress. In North America, a high level of anxiety was observed in 34.8% of women, and 43.3% showed a high level of stress. Therefore, it is very important to understand the origin of maternal stress after childbirth, and timely measures should be taken to alleviate it in order to reduce the frequency of postpartum depression [9].

There are multiple studies carried out to assess the prevalence of anxiety, stress, and depression during the postpartum period, but only a few interventional studies have been conducted to reduce the psychological distress of mothers. These include cognitive behavioural therapy (CBT), mindfulness, music, and exercise [15-18]. Although the effectiveness of interventions varies, approaches such as psychosocial, psychotherapeutic, CBT, and mind-body interventions have shown greater positive impacts on mothers who are susceptible to mental health issues and who experience high anxiety and stress during pregnancy [19, 20]. Therefore, the researcher considered a combination of back massage and affirmation relaxation to reduce the psychological distress of postpartum mothers, with a focus on those who had undergone caesarean section.

A massage stimulates the nerves and sensory receptors, which then send a message to the brain via the spinal cord and nerve pathways. The parasympathetic nervous system is activated, while the sympathetic nervous system is slowed down by the massage. It increases the circulation of blood and lymph throughout the body and also releases the neurotransmitters serotonin and endorphins, which promote positive feelings of happiness and pleasure, while reducing anxiety and depression [21, 22]. The parasympathetic nervous system calms down body activity, lowers blood pressure, and decreases the amount of sweat. It promotes the production of the hormones oxytocin and prolactin, which leads to improved breast milk production. Thus, massage helps to reduce anxiety and depression by decreasing stress during the postpartum period [23, 24]. Massage relaxes the body and helps mothers get better sleep.

Affirmation relaxation is a technique that combines deep breathing with the recitation of short, positive affirmations, according to Benson's theory of relaxation response [25, 26] and the theory of self-affirmation [27-29]. This technique can adequately shift the focus from negative emotions or stressors to positive energy and confidence, and instill hope for the future. This will reduce the stress and anxiety of mothers by improving their self-confidence [28, 29]. Considering the importance of safe and effective post-caesarean stress-relieving measures, which influence the well-being of both the mother and her newborn, the researcher carried out a study to assess the impact of affirmation relaxation and back massage on stress, anxiety, and depression in post-caesarean mothers.

## Materials and methods

The study used a quantitative research methodology with a non-randomised pretest-posttest control group design. It was conducted at Fatima Hospital, Mau, Uttar Pradesh, India, from July 1, 2022, to August 14, 2022. Twenty-four caesarean section mothers were selected through a purposive sampling technique. The investigator selected mothers admitted to the B ward after caesarean section for the experimental group, and mothers admitted to the C and E wards after caesarean section for the control group. This helped to avoid bias and blinding of the control group. In this study, the investigator intended to evaluate the effect of a combination of back massage and affirmation relaxation on depression, anxiety and stress of caesarean section mothers. The inclusion criteria included the following: mothers who delivered normal term babies by lower segment caesarean section; mothers with a single baby; mothers between 48 and 72 hours of lower segment caesarean section; and newborns with normal sucking, swallowing and rooting reflexes. The exclusion criteria included mothers who were critically ill; mothers experiencing any complications of surgery or anaesthesia; mothers with nipple or breast anomalies; mothers with postpartum psychosis; and newborns with major congenital anomalies. Data were collected using a tool with two sections: Section I consisted of a structured questionnaire on socio-personal, obstetrical and newborn data, and Section II consisted of a standardised structured questionnaire (DASS-21 (Depression, Anxiety and Stress Scale - 21 items)) to assess the depression, anxiety and stress levels of caesarean section mothers.

Procedure of data collection

After receiving approval from the institutional ethical committee of Saveetha Medical College Hospital (No.004/02/2022/IEC/SMCH dated 14/02/2022) and formal permission from the Administrator of the institution and the Head of the Department of Obstetrics and Paediatrics, orientation about the study and protocol was given to doctors and nursing staff working in the postnatal wards. The investigator met each mother, established rapport and explained the purpose and protocol of the present study. Confidentiality was ensured, and informed consent was obtained from all mothers who agreed to take part in the research. Purposive sampling was used to choose the study participants.

Data were gathered from the control group and the experimental group on the 3rd (pretest) and 6th post-operative day (post-test). Socio-personal, obstetrical, and newborn data were collected. Mothers in the experimental group were given the intervention: back massage and affirmation relaxation. The steps of the procedure were explained to each mother beforehand. First, the mother was asked to sit upright on a chair with a table in front and support the incision by holding a pillow. She was asked to breathe normally five times and then inhale deeply and slowly, hold it for two to five seconds, and exhale slowly and gently through her mouth. This was repeated five times. Then, she was asked to lean forward by keeping her head on the table. After exposing the back, gentle pressure was applied using both thumbs on either side of the spine in a circular motion between the shoulder blades. While the massage was being done, the investigator asked the mother to silently repeat simple positive affirmations such as: "I am calmer and more relaxed", "Milk production is smooth and plenty", "My baby is healthy and I am a great mother", "My baby is getting enough milk", "I get enough support from my family", and "I get enough support from my hospital staff". The whole procedure was then repeated. It was continued for 10 minutes. Just after the massage, the fullness of the breast was assessed, and the baby was put to the breast for feeding. This procedure was done four times a day for three days (7.00 a.m., 11.00 a.m., 3.00 p.m., and 7.00 p.m.). The psychological status of the mother was assessed on the 3rd day (baseline) and at 7.00 p.m. on the 6th postoperative day. The procedure was taught to the spouse or a family member at the time of discharge, and a leaflet explaining the procedure was also given for further reference. Mothers in the control group were not given any treatment except routine health assessment and health education regarding postnatal care, the importance of breastfeeding, etc. Data were collected on the 3rd and 6th post-operative days.

Analysis of data

The data were analysed using descriptive and inferential statistics with IBM SPSS Statistics for Windows, Version 26 (Released 2019; IBM Corp., Armonk, New York). Results on continuous measurements were analysed using mean and standard deviation, and categorical data were analysed using frequency and percentage distribution. A non-parametric test was used for analysis as the data were not normally distributed. The Wilcoxon signed rank test was used to assess the effectiveness of back massage and affirmation relaxation on depression, anxiety and stress before and after the intervention within the experimental and control groups. The Mann-Whitney U test was used to compare the effectiveness of the intervention between the experimental and control groups. A p-value < 0.05 was regarded as statistically significant.

## Results

As shown in Table 1, in the experimental group, 58.3% of caesarean section mothers were in the age group of 18-25 years and 41.7% of mothers were in the age group of 26-30 years, whereas in the control group, 50% of caesarean section mothers were in the age group of 18-25 years, 41.7% in the age group of 26-30 years, and 8.3% in the age group of 36-40 years. In the experimental group, 58.3% of caesarean section mothers belonged to joint families and 41.7% belonged to nuclear families, whereas in the control group, 75% belonged to joint families and 25% to nuclear families. In the experimental group, 58.4% of caesarean section mothers had an educational qualification of graduation and above, 25% had higher secondary education, 8.3% had secondary education, and 8.3% had primary education. In the control group, 41.7% had graduation and above, 33.3% had higher secondary education, 8.3% had secondary education, and 16.7% had primary education. Fisher exact test values were calculated to check the homogeneity of the groups with respect to their socio-personal variables. The values for age (0.684, p > 0.05), type of family (0.667, p > 0.05), and education (0.856, p > 0.05) show that the groups were homogeneous in terms of age, type of family, and education.

**Table 1:** Percentage and frequency distribution of socio-personal variable of caesarean section mothers in the experimental group and control group

Socio-personal Variables	Experimental Group		Control Group		Fisher Exact Test for Homogeneity
	Frequency	Percentage	Frequency	Percentage	
Age in years					
18–25	7	58.3%	5	41.7%	p = 0.684 (NS)
26–30	5	41.7%	6	50.0%	
31–35	-	-	-	-	
36–40	0	0.0%	1	8.3%	
Type of family					
Nuclear family	5	41.7%	3	25.0%	p = 0.667 (NS)
Joint family	7	58.3%	9	75.0%	
Education					
Primary	1	8.3%	2	16.7%	p = 0.856 (NS)
Secondary	1	8.3%	1	8.3%	
Higher secondary	3	25.0%	4	33.3%	
Graduate and above	7	58.3%	5	41.7%	

As displayed in Table 2, in the experimental group, 58.3% of mothers were multipara and 41.7% were primipara. In the control group, 50% of mothers were multipara and 50% were primipara. In the experimental group, 58.3% of mothers had undergone elective LSCS and 41.7% had undergone emergency LSCS. In the control group, 66.7% of mothers had undergone emergency LSCS and 33.3% had undergone elective LSCS. In the experimental group, 66.7% of newborns were girls and 33.3% were boys. In the control group, 50% of newborns were boys and 50% were girls. In the experimental group, 75% of newborns started breastfeeding after four hours and 25% started breastfeeding within two to four hours. In the control group, 83.3% of newborns started breastfeeding after four hours and 16.7% started breastfeeding within two to four hours. Fisher exact test values were calculated to check the homogeneity of the groups with respect to their obstetrical and newborn variables. The p-values for parity (1.000, p > 0.05), type of LSCS (0.417, p > 0.05), gender (0.680, p > 0.05), and timing of initiation of breastfeeding after caesarean section (1.000, p > 0.05) show that the groups were homogeneous in terms of parity, type of LSCS, gender, and timing of initiation of breastfeeding after caesarean section.

**Table 2:** Percentage and frequency distribution of newborn and obstetrical variables of caesarean section mothers in the experimental and control group LSCS: lower segment cesarean section, NS: not significant.

Obstetric and Newborn Variables	Experimental Group		Control Group		Fisher Exact Test for Homogeneity
	Frequency	Percentage	Frequency	Percentage	
Parity					
Primipara	5	41.7%	6	50.0%	1.000 (NS)
Multi para	7	58.3%	6	50.0%	
Type of LSCS					
Elective	7	58.3%	4	33.3%	0.414 (NS)
Emergency	5	41.7%	8	66.7%	
Gender					
Male	4	33.3%	6	50.0%	0.680 (NS)
Female	8	66.7%	6	50.0%	
Birth weight of the newborn					
2500–3000 g	7	58.3%	9	75.0%	0.371 (NS)
3001–3500 g	5	41.7%	2	16.7%	
3501–4000 g	0	0.0%	1	8.3%	
Timing of initiation of breastfeeding after caesarean section					
Within 3 minutes	-	-	-	-	1.000 (NS)
Between 30 minutes and 1 hour	-	-	-	-	
Between 1 and 2 hours	-	-	-	-	
Between 2 and 4 hours	3	25.0%	2	16.7%	
After 4 hours	9	75.0%	10	83.3%	

As displayed in Table 3, in both the experimental and control groups, before the intervention on day 3, 66.67% of mothers were in the normal level and 33.3% were in the mild level of depression. On day 6, after the intervention, in the experimental group, 91.67% of mothers were in the normal level and 8.33% were in the mild level of depression. In the control group, 83.3% of mothers were in the normal level and 16.67% were in the mild level of depression on day 6.

**Table 3:** Percentage and frequency distribution of depression among caesarean section mothers before and after intervention in the experimental and control groups

Depression	Experimental Group				Control Group			
	Before Intervention		After Intervention		Before Intervention		After Intervention	
	f	%	f	%	f	%	f	%
Normal (0–9)	8	66.67	11	91.67	8	66.67	10	83.33
Mild (10–13)	4	33.33	1	8.33	3	25.00	2	16.67
Moderate (14–20)	0	0.00	0	0.00	1	8.33	0	0.00
Severe (21–27)	-	-	-	-	-	-	-	-
Extremely severe (28+)	-	-	-	-	-	-	-	-

In the experimental group, as shown in Table 4, before the intervention on day 3, 8.33% of mothers were at a normal level of anxiety, 16.7% at a mild level, 66.66% at a moderate level, and 8.33% at a severe level of anxiety. In the control group on day 3, before the intervention, 33.33% of mothers were at a mild level, 58.3% at a moderate level, and 8.33% at a severe level of anxiety. On day 6, after the intervention, in the experimental group, 50% of mothers were at a normal level of anxiety, and 25% each were at mild and moderate levels. In the control group on day 6, 16.67% of mothers were at a normal level of anxiety, 25% at a mild level, and 58.33% at a moderate level. 

**Table 4:** Percentage and frequency distribution of anxiety of caesarean section mothers before and after intervention in the experimental and control groups

Anxiety	Experimental Group				Control Group			
	Pre-test		Post-test		Pre-test		Post-test	
	f	%	f	%	f	%	f	%
Normal (0-7)	1	8.33	6	50.00	0	0.00	2	16.67
Mild (8-9)	2	16.67	3	25.00	4	33.33	3	25.00
Moderate (10-14)	8	66.67	3	25.00	7	58.33	7	58.33
Severe (15-19)	1	8.33	0	0.00	1	8.33	0	0.00
Extremely severe (20+)	-	-	-	-	-	-	-	-

As shown in Table 5, in both the experimental and control groups, before the intervention on day 3, 91.66% of mothers had a normal level of stress and 8.3% had a mild level of stress. On day 6, in both the experimental and control groups, all (100%) mothers had a normal level of stress.

**Table 5:** Percentage and frequency distribution of stress of caesarean section mothers before and after intervention in the experimental and control groups

Stress	Experimental Group				Control Group			
	Pre-test		Post-test		Pre-test		Post-test	
	F	%	f	%	f	%	f	%
Normal (0-14)	11	91.67	12	100.00	11	91.67	12	100.00
Mild (15-18)	1	8.33	0	0.00	1	8.33	0	0.00
Moderate (19-25)	0	0.00	0	0.00	0	0.00	0	0.00
Severe (26-33)	-	-	-	-	-			
Extremely severe (34+)	-	-	-	-	-	-		

Table 6 shows that the mean depression score was reduced from 8.00 ± 2.08 to 6.50 ± 1.93, the mean anxiety score was reduced from 11.67 ± 3.06 to 6.50 ± 3.53, and the mean stress score was reduced from 10.33 ± 3.17 to 6.00 ± 2.83 after the intervention in the experimental group. The z-values obtained for depression, anxiety, and stress in the experimental group differed significantly after the intervention at p < 0.05, and it is inferred that the combination of back massage and affirmation relaxation had a significant effect on depression, anxiety, and stress in caesarean section mothers.

**Table 6:** Comparison of means in depression, anxiety, and stress of mothers in experimental group after intervention

Variables	Before Intervention		After Intervention		Wilcoxon Signed Rank Test (Z)	p-value
	Mean	SD	Mean	SD		
Depression	8.00	2.08	6.50	1.93	-2.50	0.013
Anxiety	11.67	3.06	6.50	3.53	-2.95	0.003
Stress	10.33	3.17	6.00	2.83	-3.12	0.002

As displayed in Table 7, the U value of 35.0 (p = 0.033) obtained for stress after the intervention was statistically significant at the 0.05 level. The U value of 38.5 (p = 0.052) obtained for anxiety was nearly statistically significant at the 0.05 level of significance. Hence, it was inferred that the combination of back massage and affirmation relaxation was effective in reducing stress and anxiety among mothers who had undergone caesarean section. The depression score was not statistically significant, which may be due to the limited sample size.

**Table 7:** Mean, standard deviation, and U-value showing the impact of back massage and affirmation relaxation on depression, anxiety, and stress among caesarean section mothers between experimental and control groups

Variables		Experimental		Control		Mann Whitney U Test	
		Mean	SD	Mean	SD	U value	p-value
Depression	Before intervention	8.00	2.09	8.50	2.28	63.5	0.630
	After intervention	6.50	1.93	7.00	2.49	65.0	0.713
Anxiety	Before intervention	11.67	3.06	11.50	2.97	70.0	0.932
	After intervention	6.50	3.53	9.17	1.99	38.5	0.052
Stress	Before intervention	10.33	3.17	11.00	2.63	62.5	0.590
	After intervention	6.00	2.83	8.67	2.31	35.0	0.033

## Discussion

The present study attempted to assess the impact of back massage and affirmation relaxation on the psychological status of caesarean section mothers. The findings revealed an overall decrease in depression, anxiety, and stress among caesarean section mothers in the experimental group compared to the control group. The Wilcoxon signed rank test showed a statistically significant reduction in depression, anxiety, and stress at p < 0.05 among mothers in the experimental group after the intervention. The Mann-Whitney test showed a statistically significant decrease in stress and a nearly significant reduction in anxiety between the experimental and control groups after the intervention at p < 0.05, indicating that back massage and affirmation relaxation were effective in improving the psychological status of caesarean section mothers.

The findings of another randomised controlled trial study with 76 participants, conducted on a self-empowerment affirmation relaxation programme for postpartum mothers with "baby blues", also support the current study. It demonstrated that there was a significant reduction in postpartum blue scores between the intervention and control groups at the first, second and third month follow-up (p = 0.01, 0.001 and 0.002, respectively) [30].

The results of the current investigation are supported by the findings of a pilot study on the effect of Benson’s relaxation therapy on post-caesarean pain and stress in Gujarat. In this study, the mean pain score decreased from 7.00 to 2.20 among mothers in the experimental group, and from 7.60 to 4.60 in the control group. The mean stress score was reduced from 90.60 to 57.60 among experimental group mothers, and from 92.00 to 75.20 among those in the control group [31].

The findings of the present study were consistent with those of a study conducted among Iranian postpartum mothers who had delivered via normal vaginal delivery, which demonstrated a significant decrease in anxiety level following the intervention (37.32 ± 9.01 vs 30.82 ± 6.22, p ≤ 0.001) and the morning after intervention (37.40 ± 8.29 vs 30.66 ± 7.19, p ≤ 0.001) in the experimental group compared to the control group [32].

The findings of the present study are also congruent with the results of an analytical experimental study with a randomised controlled trial, which showed that the anxiety scale in the treatment group (median = 24, SD = 4.45) was lower than in the control group (median = 34, SD = 6.93), and the difference was statistically significant (p < 0.001) [33].

The findings of the present study are in line with the results of earlier research and suggest the success of the intervention, combining back massage and affirmation relaxation, in improving the psychological well-being of caesarean mothers by reducing depression, anxiety, and stress.

It is important to take into account a few limitations when evaluating the findings. Firstly, the scale used, DASS-21, was a self-report tool intended to evaluate symptoms associated with stress, anxiety, and depression. It was not specifically designed for new mothers. Therefore, other instruments developed for postnatal women should be considered for evaluating psychological distress in this population. Secondly, to more reliably reflect the impact of the intervention, a more objective approach needs to be employed. Thirdly, due to the small sample size, the study's findings cannot be generalised to all postnatal mothers who have undergone caesarean sections.

## Conclusions

Combination therapy is always more successful at managing patients' psychological distress when compared to a single intervention. Our findings showed that, compared to caesarean mothers who did not receive the intervention, mothers who had the back massage and affirmation relaxation experienced less psychological distress. The technique significantly improved the comfort and confidence of women who had caesarean sections. Nurses play a significant role in promoting the physical and mental health of postpartum mothers. For mothers who gave birth either naturally or by caesarean section, back massage and affirmation relaxation should be part of the standard postpartum care offered by hospitals. During postnatal follow-up visits, community health nurses and other peripheral healthcare providers can readily teach and disseminate these approaches to the spouses or caretakers. For mothers who have had caesarean sections, it ought to be viewed as a therapeutic modality to support their recovery and prevent psychological discomfort. It is recommended that, during student nurses' training, these kinds of techniques be included in the curriculum. The study concludes that a combination of affirmation relaxation and back massage is a cost-effective, non-invasive and patient-friendly method for enhancing the psychological well-being of women who have undergone caesarean sections in the early postpartum phase.
